# The effect of *Annona muricata* (Graviola) on the prevention of brain damage due to ionizing radiation in rats

**DOI:** 10.1016/j.heliyon.2024.e25932

**Published:** 2024-02-08

**Authors:** Ozlem Elmas, Emrah Keskin, Havva Hande Keser Sahin, Berrak Guven, Ghada Almisned, Hesham M.H. Zakaly, H.O. Tekin, Antoaneta Ene

**Affiliations:** a Department of Radiation Oncology, Bulent Ecevit University Practice and Research Hospital, Zonguldak, Turkey; b Department of Neurosurgery, Bulent Ecevit University Practice and Research Hospital, Zonguldak, Turkey; c Department of Pathology, University Corum Training and Research Hospital, Corum, Turkey; d Department of Biochemistry, Bulent Ecevit University Practice and Research Hospital, Zonguldak, Turkey; e Department of Physics, College of Science, Princess Nourah Bint Abdulrahman University, P.O. Box 84428, Riyadh 11671, Saudi Arabia; f Institute of Physics and Technology, Ural Federal University, Yekaterinburg, 620002, Russia; g Physics Department, Faculty of Science, Al-Azhar University, Assiut, Egypt; h Department of Medical Diagnostic Imaging, College of Health Sciences, University of Sharjah, 27272, Sharjah, United Arab Emirates; i Istinye University, Faculty of Engineering and Natural Sciences, Computer Engineering Department, Istanbul, 34396, Turkey; j INPOLDE Research Center, Department of Chemistry, Physics and Environment, Faculty of Sciences and Environment, Dunarea de Jos University of Galati, 47 Domneasca Street, 800008 Galati, Romania

**Keywords:** *radiation*, *Ionizing*, *Annona muricata*, *Acute brain injuries*, *Preventative care*

## Abstract

In this study, it was aimed to evaluate the effect of ethanol extract of *Annona Muricata* (AM) leaves in the prevention of brain damage caused by ionizing radiation (IR). This study was conducted in the Experimental Animal Research Unit of a university with 28 adults female Wistar Albino rats. The experimental groups were as follows: Control group (n = 8), AM group (n = 6), IR group (n = 8), AM + IR group (n = 6). In the IR group, astrocyte hypertrophy, microglial reaction and inflammatory reaction levels were significantly higher than the control and AM groups (*P* < 0.001). Edema was significantly higher in the IR group compared to the control group (*P=*0.001). The MDA of the IR group was significantly higher compared to the control group and AM group (*P=*0.031, *P=*0.006, respectively). The MDA of the AM + IR group was significantly higher than the AM group (*P=*0.039). Our findings show that histomorphology and oxidant damage caused by IR can be ameliorated using AM, as demonstrated by the comparison of the controls to AM + IR recipients, which showed similar histomorphology and oxidant damage levels.

## 1 Introduction

The utilization of radiation for diagnostic and therapeutic purposes involves both internal and external irradiations implementing electromagnetic radiation such as X-rays and gamma-rays. The ionizing ability of radiation, which is administered to patients both internally and externally, has the potential to harm healthy tissues and cause significant damage. One of the applications addressed, namely radiotherapy, deals with the structural degradation of tumors through the utilization of external LINAC-generated X-rays that are directed towards the patient at very high energy levels. Aside from the beneficial impacts of radiotherapy interventions, it is essential to prioritize the identification and mitigation of potential hazards that could compromise patient safety. Exposure of the whole body to ionizing radiation (IR) causes overproduction of reactive oxidative species (ROS) through molecular ionization and, consequently, an increase in cellular oxidative stress. It has been shown that multiple organ dysfunction can be triggered through such effects [1,2]. The brain is among the organs that is sensitive to IR exposure and can undergo serious changes, including demyelination, necrosis and vascular problems [3]. In addition, neurogenesis deficiency, vascular problems and dysfunction of the blood-brain barrier can be seen due to chronic inflammation and increased ROS levels. IR-induced brain damage seriously affects cognitive functions and reduces quality of life [4]. Therefore, it is important to search for effective interventions in preventing these undesirable effects. Meanwhile, natural antioxidants have been extensively researched for their radioprotective properties. Among them, *Annona Muricata* (AM), commonly known as Graviola (or soursop, guanabana), is a plant that grows in countries with tropical climates [5]. The leaves of the AM plant have many active substances including alkaloids and phenols; however, the most dominant are acetogens [6]. The intended use of Graviola leaves varies greatly from region to region, yet it is commonly utilized as a traditional medicine, especially in Africa and South America. It has been shown to have anticonvulsant, antiparasitic, antiarthritic, antimalarial, antidiabetic, hepatoprotective and anticancer effects [[7], [8], [9]]. Although reported less frequently, its properties are also suggested to include antifungal, antibacterial and antioxidant effects [7]. Although the anticancer and antioxidant properties of the components of the AM leaf have been demonstrated in many studies, the number of studies investigating AM for its effect in preventing IR-induced damage caused by cancer treatments is quite limited. In this study, it was aimed to evaluate the effect of ethanol extract of AM leaves in the prevention of brain damage caused by IR. The results obtained from the current investigation have the potential to be useful in enhancing comprehension of the currently available radioprotective substances and their potential use in future research efforts.

## 2 Materials and methods

### 2.1 Animals

**Fig. 1:**
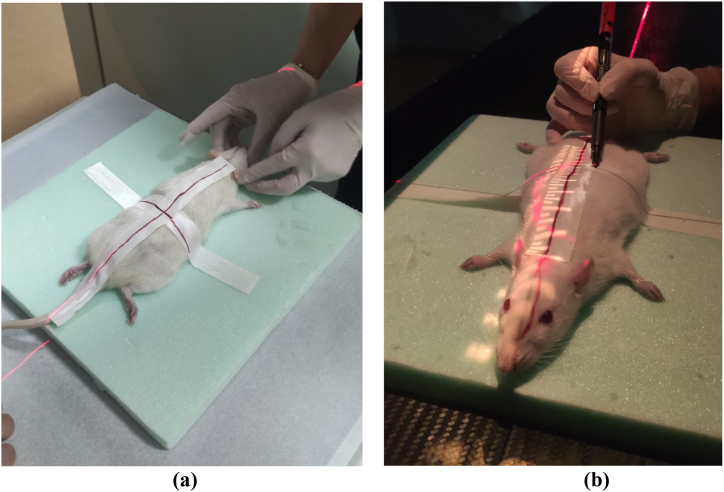
**(a)** Preparation of rat **(b)** marking of the rat before radiation exposure.

The present investigation received ethical approval from the Clinical Research Ethics Committee of Zonguldak Bülent Ecevit University, with the assigned approval number **2020/05** and the date of approval being **June 04, 2020**. Our research was conducted at the Experimental Animal Research Unit of the Faculty of Medicine at Zonguldak Bülent Ecevit University through 28 adults female Wistar Albino rats (see Fig. 1a). To assure comparable biological and physiological characteristics, rats were divided into four groups: one control group and three experimental groups. Throughout the duration of the investigation, all animals were fed ad libitum potable water and enriched rodent diet containing 21% crude protein under ideal laboratory conditions (22 ± 1 °C, 50–55% humidity, 12 h light/dark cycle).

### 2.2 Experimental design, irradiation, and *Annona muricata* extraction procedure

Prior to the irradiation protocols conducted at the Radiation Oncology Department of Zonguldak Bulent Ecevit University School of Medicine, all animals, except for the control group, were intraperitoneally administered a dosage of 90 mg/kg ketamine (Ketalar-Eczacbaşi/Turkey) and 10 mg/kg xylazine (Rompun-Bayer/Turkey). Based on prior research conducted on rodent models [10], the appropriate amount of ionizing radiation (IR) to induce radiation-induced damage was determined. The marking procedure was performed on rats (see Fig. 1b). Using a linear accelerator, 20 Gy of X-rays were administered in a single fraction to the entire body of rodents in the supine position. The extract obtained from the dried leaves of the AM plant by ethanol-based filtration was administered to the animals as the active ingredient. Meanwhile, we designed the experimental groups as follows.

• Group-I (n = 8) (Control-Group): daily gavage administration of 0.01 mL/kg of distilled water for 9 days.
• Group-II (n = 6) (AM-Group) administrated 300 mg/kg of AM by gavage once daily for 9 days. All rodents were sacrificed 96 h after the completion of radiotherapy procedures.
• Group-III (n = 8) (IR group): 0.01 mL/kg of distilled water was administered via gavage daily for nine days. Radiation to the entire body (20 Gy) was administered 1 h after the last gavage.
• Group-IV (n = 6) (AM + IR group) administrated 300 mg/kg of Graviola leaf extract by gavage once daily for 9 days.

Meanwhile, radiation to the entire body (20 Gy) was administered 1 h after the last gavage. Dose calculation was carried out on 16 rats (approximately 200g)-300 mg/kg-9 days->16 × 60 mg x9 days = 8.640 mg. In this study, Graviola (*Annona muricata* L.) leaves that had been air dried were subjected to a washing process using distilled water. Subsequently, small 25g leaf samples were extracted in a solution consisting of a 50:50 mixture of ethanol for a duration of 7 days. The ethanol was removed from the extract using a rotary evaporator device (Heidolph, Germany). Subsequently, the extract underwent lyophilization using a Telstar-LyoQuest apparatus and was thereafter stored overnight to get the dry extract. The desiccated extract was stored at a temperature of −20 °C until it was utilized.

### 2.3 Histopathological evaluation

A group of 28 brain tissues was subjected to fixation in a 10% formalin solution for a period of 12 h, after which they were subsequently embedded in paraffin blocks. The specimens underwent a sectioning process, resulting in a thickness of 4 μm, followed by the application of hematoxylin and eosin staining. The study encompassed the analysis of seven criteria, specifically astrocyte hypertrophy, microglial reactivity, inflammatory reaction, vascular telangiectasia, endothelial expansion, edema, and axonal damage in brain tissue. These characteristics were assessed across four unique groups. The histomorphology properties of the tissues and cells were assessed. The assessment of each criterion spanned from 0 to 3, with 0 representing the absence of harm, 1 indicating minimal damage, 2 denoting moderate damage, and 3 representing significant damage.

### 2.4 Immunohistochemical evaluation

The association between hypertrophy and the expression of glial fibrillary acidic protein (GFAP) has been demonstrated [11]. The immunohistochemical analysis involved incubating sections with Polyclonal Rabbit Anti-Glial Fibrillary Acidic Protein (DAKO, Glostrup, Denmark).

### 2.5 Biochemical analysis

The tissues underwent two rounds of cold saline solution washing, were subsequently deposited into labeled glass containers, and were preserved in a deep freezer at a temperature of −80 °C until further processing. The tissue samples were subjected to homogenization using a glass Teflon homogenizer (Ultra Turrax IKA T18 Basic) with phosphate buffered saline (pH 7.4). The homogenates underwent centrifugation at a force of 5000 times the acceleration due to gravity at a temperature of 4 °C for a duration of 15 min, following which the resulting supernatants were promptly subjected to analysis. The quantification of Malondialdehyde (MDA) was conducted using a commercially available Enzyme-Linked Immunosorbent Assay (ELISA) kit obtained from Cloud-Clone Corporation located in Wuhan, China. The ABTS-based method was utilized to measure the trolox equivalent antioxidant capacity assay (TAS estimation), as conducted by Cloud-Clone Corp located in Wuhan, China. The outcomes were documented on a per milligram protein basis of the tissue.

### 2.6 Statistical analysis

The statistical analyses were performed utilizing the SPSS v21 software (SPSS Inc., Chicago, IL, USA). The normality assessment was conducted using the Shapiro-Wilk test. The data provided is displayed in two distinct formats for continuous variables. These formats include mean ± standard deviation or median (1st quartile – 3rd quartile), and the choice of format is dependent on the normality of distribution. The presentation of categorical variables is in the form of frequency (percentage). The researchers employed the one-way ANOVA test to examine variables that exhibited a normal distribution. The post-hoc Tukey test was utilized to determine the presence or absence of statistically significant differences. The Kruskal-Wallis test was utilized to examine variables that exhibited non-normal distribution. The application of the Bonferroni correction technique was employed to carry out pairwise comparisons. The categorical variables were evaluated using the Pearson's Chi-square test. Results were deemed statistically significant if the **p-value** was below 0.05.

## 3 Results

**Fig. 2:**
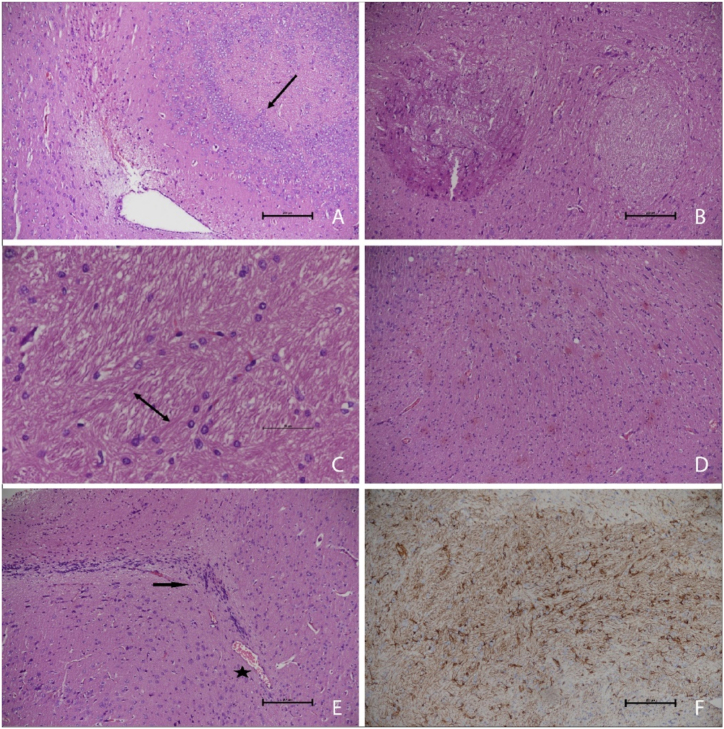
**(A**–**F).** Effects of radiation on brain cortex in IR group. (**A)** Astrocytes showed diffuse hypertrophy (arrow), vascular dilatation, edema (H&E, X10) **(B)** Axonal damage (H&E, X10) **(C)** Prominence of Rosenthal fibers, (arrow) (H&E, X10) **(D)** Microglial reaction (H&E, X10) **E)** Inflammatory reaction (arrow), vascular dilatation, edema (asterisk) (H&E, X10) **(F)** Glial fibrillary acidic protein, (H&E, X10).

The present study examined many parameters, such as hypertrophy, microglial reactivity, inflammatory response, vascular ectasia, endothelial enlargement, edema, and axonal injury, within astrocytes. The astrocytes in the IR group had diffuse hypertrophy, an increase in number, and slight clustering of nuclear chromatin. In contrast, the brain tissues of the control and AM groups demonstrated normal characteristics. The activation of microglial cells and the manifestation of inflammation were shown to be common, with a prevailing inflammatory reaction, specifically involving lymphocytes, being documented. The endothelial cells demonstrated notable vascular dilation at a localized level. The prevalence of edema was shown to be high, especially in close proximity to the veins, and had a higher degree of severity. The presence of prominent Rosenthal fibrils was detected. Astrocytes in the IR + AM group exhibited mild hypertrophy, and the disappearance of chromatin clusters was noted. The microglial cells' response and the consequent inflammatory process were characterized by a relatively mild nature. The determination of notable levels of vascular dilation and endothelial damage was not established. Both the Control and AM groups displayed conventional histomorphological structure in their cerebral cortex. The group studying IR observed significant increases in astrocyte numbers, microglial cell response, diffuse hypertrophy in nuclear chromatin, inflammatory cell response, vascular telangiectasia, and edema. The graphical representation presented in Fig. 2 (A-F) illustrates an increase in the quantity of Rosenthal fibrils. The IR group exhibited a statistically significant increase in astrocyte hypertrophy, microglial reaction, and inflammatory reaction in comparison to the control and AM groups (P < 0.001 for each).

Furthermore, the study revealed a statistically significant increase in edema among the intervention group as compared to the control group (p = 0.001), as presented in Table 1. The study found a significant difference in MDA values between the groups (P = 0.003), but no significant difference in TAS values (p = 0.118). The study found that the MDA value of the group exposed to infrared radiation was significantly greater than that of both the control group and the group exposed to AM radiation (p = 0.031, p = 0.006, respectively). The study found that the MDA value of the group treated with AM and IR was significantly greater than that of the group treated with AM alone (p = 0.039), as shown in Table 2.

**Table 1:** Histomorphological findings according to treatment groups.

	Treatment groups				
	Control (n = 8)	AM (n = 6)	IR (n = 8)	AM + IR (n = 6)	p
Hypertrophy in astrocytes	0.5 (0–1)	0 (0–1)	2.5 (1.5–3)^a,b^	2 (1–2)	0.001
Normal	4 (50%)	4 (66.7%)	0 (0%)	0 (0%)	0.008
Light	4 (50%)	2 (33.3%)	2 (25%)	2 (33.3%)	
Medium	0 (0%)	0 (0%)	2 (25%)	3 (50%)	
Heavy	0 (0%)	0 (0%)	4 (50%)	1 (16.7%)	
Microglial reaction	1 (0.5–1)	1 (1–1)	3 (2–3)^a,b^	2 (1–2)	<0.001
Normal	2 (25%)	1 (16.7%)	0 (0%)	0 (0%)	0.001
Light	6 (75%)	5 (83.3%)	1 (12.5%)	2 (33.3%)	
Medium	0 (0%)	0 (0%)	2 (25%)	4 (66.7%)	
Inflammatory reaction	1 (0–1)	1 (0–1)	2 (2 - 2)^a,b^	1 (1–1)	0.001
Normal	3 (37.5%)	2 (33.3%)	0 (0%)	0 (0%)	<0.001
Light	5 (62.5%)	3 (50%)	1 (12.5%)	6 (100%)	
Medium	0 (0%)	1 (16.7%)	7 (87.5%)	0 (0%)	
Vascular telengiectasis	0 (0–0)	0 (0–0)	1 (0–1)	0 (0–0)	0.146
Normal	7 (87.5%)	5 (83.3%)	3 (37.5%)	5 (83.3%)	0.059
Light	1 (12.5%)	1 (16.7%)	5 (62.5%)	0 (0%)	
Medium	0 (0%)	0 (0%)	0 (0%)	1 (16.7%)	
Endothelial enlargement	0 (0–0)	0 (0–0)	0 (0–0)	0 (0–0)	0.475
Normal	8 (100%)	6 (100%)	7 (87.5%)	6 (100%)	0.459
Light	0 (0%)	0 (0%)	1 (12.5%)	0 (0%)	
Edema	0 (0–0)	1 (0–2)	2 (2 - 2)^a^	0.5 (0–1)	0.001
Normal	8 (100%)	3 (50%)	0 (0%)	3 (50%)	0.002
Light	0 (0%)	0 (0%)	1 (12.5%)	2 (33.3%)	
Medium	0 (0%)	3 (50%)	7 (87.5%)	1 (16.7%)	
Axonal damage	0 (0–0)	0 (0–0)	0 (0–0)	0.5 (0–1)	0.306
Normal	7 (87.5%)	5 (83.3%)	7 (87.5%)	3 (50%)	0.290
Light	1 (12.5%)	1 (16.7%)	1 (12.5%)	3 (50%)	

Data were given as median (1st quartile - 3rd quartile) for continuous variables and as frequency (percentage) for categorical variables. ^a, b^ Significantly different from the control group and AM treated group, respectively.

AM: *Annona muricata*, IR: irradiation.

**Table 2:** MDA and TAS levels according to treatment groups.

Level	Treatment groups				
	Control	AM	IR	AM + IR	p
MDA (ng/mg protein)	35.69 ± 3.80	32.25 ± 8.84	46.14 ± 7.36^a,b^	43.92 ± 7.94 ^b^	0.003
TAS (mM Trolox/mg protein)	1.16 ± 0.43	1.10 ± 0.40	1.55 ± 0.26	1.53 ± 0.60	0.118

Data were given as mean ± standard deviation. ^a, b^ Significantly different from the control group and AM treated group, respectively.

AM: *Annona muricata*, IR: irradiation, MDA: malondialdehyde, TAS: total antioxidant status.

## 4 Discussion

**Fig. 3:**
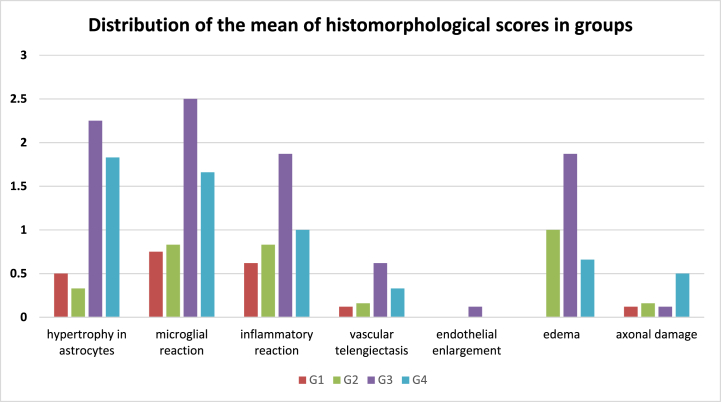
Distribution of the mean of histomorphological scores in groups.

Ionizing radiation, which is the main component of radiotherapy, causes various degrees of damage to many tissues in the body. In this study, we assessed the effect of AM in preventing ionized radiation-induced brain damage. It was found that hypertrophy in astrocytes, microglial reaction, inflammatory reaction, and MDA levels were higher in animals given IR compared to the control and AM groups (see Fig. 3). There was no significant difference between the control group and AM + IR groups in terms of these variables, indicating an ameliorative effect of AM on IR-induced injury. Although various therapeutic and protective properties of AM were examined in previous studies, the number of studies examining the effect of AM on the secondary inflammatory response induced by radiotherapy and oxidative damage caused by ROS is limited. Among these studies, there is no study examining the effect of AM on brain tissue. In a study examining the level of IR-induced oxidative damage in kidney and liver tissues and the radioprotective effect of AM, Mansour et al. found that AM administration prior to IR significantly reduced MDA levels compared to those that had received only IR (6 Gy). In addition, they showed that when AM was applied before IR, the level of DNA fragmentation decreased and the activity of caspase 3 and superoxide dismutase (SOD) increased significantly. As a result, they claimed that AM is radioprotective thanks to its antioxidant properties [12]. Many different studies have emphasized the antioxidant effect of AM, as well as its ROS-decreasing effect and thus its radioprotective effect [5,13]. George et al. showed that the radioprotective effect of AM is due to its DNA-protective effect and its oxidative damage-reducing effect [14]. In a study examining the effect of AM application on reducing IR-induced skin tissue damage, Byun et al. showed that AM application before IR increased cell viability, decreased apoptosis, decreased inflammatory response and damage, and increased SOD and catalase activity. As a result of the study, they suggested that AM reduces radiation-induced skin damage by increasing antioxidant enzyme activity and reducing inflammation in normal human epidermal keratinocytes cells [15]. The results of these studies underline the antioxidative effects of AM. In a study that assessed the protective effect of AM in IR-induced liver damage (via Serum Glutamic Pyruvate Transaminase measurement), Adelia et al. showed that the decrease in liver damage with AM was dose-dependent [16]. Consistent with all previous studies, it has been shown in our study that AM has radioprotective properties. In our study, in addition to the changes in micromorphological findings in the IR group, the MDA level, which is an important indicator of oxidative stress, was found to be significantly higher in the IR group compared to the AM + IR group. Consistent with our results, in a study investigating the cellular effects of IR, Liu et al. reported that IR affects antioxidant enzymes, causing an increase in MDA level and ultimately leading to negative consequences in cellular functions [17]. In a study investigating the effect of oral AM administration on tumor size for 30 days prior to low dose IR (2 Gy) application, El-Tawiil et al. reported that combining treatment is more effective in limiting tumor proliferation and reducing tumor size [18]. It can be stated that AM administration before or with IR can both reduce IR-induced damage and is effective in limiting tumor size. The mechanism of the protective effect of AM against IR is not clear. Studies have shown that IR-induced damage is caused by the increase in free radicals that attack DNA, lipids, and proteins, demonstrating that IR induces severe cellular damage [19,20]. The study has demonstrated that AM leaves' water and methanol-based extracts exhibit protective effects on DNA against cellular toxicity induced by H_2_O_2_. The effects were measured through various tests, including the 2,2-diphenyl-1-picrylhydrazyl (DPPH) radical scavenging assay, ferric reducing antioxidant property, and hydroxyl radical scavenging activity tests [14]. According to sources, the plant's seeds and leaves possess enzymatic antioxidants, including catalase and SOD, as well as non-enzymatic antioxidants, such as vitamin C and vitamin E [21]. In a study conducted by Padma and colleagues, it was demonstrated that the application of ethanol extracts derived from the rootbark of AM resulted in a reduction of lipid peroxidation caused by cold immobilization stress in both brain and liver tissues of rats [22,23]. The study revealed that the administration of AM root-bark extract at a dosage of 200 mg/kg exhibited a protective impact against carbon tetrachloride-induced oxidative stress in rats. This effect could be attributed to the potential elevation of antioxidant levels. A study conducted using the DPPH test has also exhibited the antioxidant activity of the root-bark extract of AM [24]. Laksmitawati et al. conducted an in vitro investigation to ascertain the mechanism by which AM leaf extract exerts its anti-inflammatory properties. The study revealed that the extract inhibits the production of inflammatory mediators, including TNF-α, IL-1β, IL-6, and nitric oxide (NO). The research findings indicate that the application of 50 μg/mL AM leaf extract resulted in a significant reduction of TNF-α level by 47%, as well as a reduction of IL-6 and NO levels by 64%, as reported in Ref. [25]. In previous research conducted by Moghadamtous et al., the impact of AM leaf extract on wound healing in rats was investigated. The findings revealed that the anti-inflammatory properties of the extract were activated through the upregulation of HSP70 [5]. Several in vitro models have demonstrated that the leaves of AM exhibit a more potent antioxidant effect in comparison to numerous other natural plant species [26]. The aforementioned data strongly suggest the potential utilization of AM as a natural source of antioxidants.

## 5 Conclusions

One of the primary objectives in the field of radiation applications, alongside the therapeutic advantages, is to carefully consider and implement preventive measures in situations that might potentially pose risks to the patient. The research investigates the potential benefits of optimizing planning, examining various gadget features, and exploring other types of alternative extracts in order to facilitate risk reduction actions. The radioprotective characteristics of *Annona Muricata* (AM), which stand out with their intriguing features and have previously been examined by our study group [27,28] for other organs, in the very radiosensitive brain, are those which spurred this research. The principal constraint of our investigation is the restricted analysis of biochemical and histopathological parameters. Nonetheless, this is a common occurrence in studies that concentrate on this subject matter. Furthermore, it should be noted that the current study has identified significant changes. However, it is important to acknowledge that the limited number of animals used in the experiment is a constraint that is inherently associated with animal studies of this nature. The study's findings indicate the existence of significant histomorphology abnormalities, including astrocyte hypertrophy, microglial reaction, and inflammatory response, as well as increased MDA levels indicative of oxidant damage, in the brain tissue of the IR group relative to the control group. In summary, these results demonstrate the presence of severe pathological changes in the IR group's brain tissue. The application of AM prior to IR (AM + IR group) resulted in histomorphology findings and oxidant damage levels that were comparable to those of the control group, suggesting that the use of AM was effective in mitigating IR-induced damage. The incorporation of AM into radiotherapy regimens warrants consideration as a potential strategy for mitigating treatment-associated adverse events.

## Ethics declarations

The Zonguldak Bülent Ecevit University Clinical Research Ethics Committee issued the ethical approval (**No: 2020/05, Date: June 04, 2020**).

## Funding

Princess Nourah bint Abdulrahman University Researchers Supporting Project number (**PNURSP2024R149**), Princess Nourah bint Abdulrahman University, Riyadh, Saudi Arabia**.**

## Data Availability

Data will be made available on request.

## CRediT authorship contribution statement

**Ozlem Elmas:** Conceptualization, Data curation, Formal analysis, Investigation, Methodology, Writing – original draft, Resources. **Emrah Keskin:** Investigation, Methodology, Validation, Writing – original draft. **Havva Hande Keser Sahin:** Formal analysis, Investigation, Methodology, Writing – original draft. **Berrak Guven:** Investigation, Methodology, Validation, Writing – original draft. **Ghada Almisned:** Funding acquisition, Investigation, Visualization, Writing – original draft. **Hesham M.H. Zakaly:** Investigation, Methodology, Validation, Writing – original draft. **H.O. Tekin:** Formal analysis, Investigation, Methodology, Software, Writing – original draft, Writing – review & editing. **Antoaneta Ene:** Data curation, Methodology, Project administration, Software, Visualization, Writing – review & editing, Supervision.

## Declaration of competing Interest

The authors declare that they have no known competing financial interests or personal relationships that could have appeared to influence the work reported in this paper.
